# The Role of Cognitive Deficits in Borderline Personality Disorder with Early Traumas: A Mediation Analysis

**DOI:** 10.3390/jcm12030787

**Published:** 2023-01-18

**Authors:** Paola Bozzatello, Cecilia Blua, Claudio Brasso, Paola Rocca, Silvio Bellino

**Affiliations:** Department of Neuroscience, University of Turin, Via Cherasco 15, 10126 Turin, Italy

**Keywords:** executive functions, personality disorders, social cognition, BPD psychopathology

## Abstract

(1) Background: although studies of cognitive functions are still limited in borderline personality disorder (BPD), the initial evidence suggested that BPD patients have deficits of executive functions and social cognition. In addition, patients who report physical and psychic traumatic experiences in childhood and adolescence show considerable neurocognitive impairment and severe BPD symptoms. The present study has a twofold aim: (1) to evaluate the differences in neurocognitive performances between BPD patients and healthy controls and (2) to verify in the BPD patients group whether neurocognitive deficits have the role of mediating the effect of early traumas on BPD psychopathology. (2) Methods: 69 subjects were enrolled: 38 outpatients with a diagnosis of BPD (DSM-5) and 31 healthy controls. BPD patients were tested with the Borderline Personality Disorder Severity Index (BPDSI), and the Childhood Trauma Questionnaire–Short Form (CTQ-SF). All subjects were evaluated with the Iowa Gambling task (IGT), the Berg card sorting test (BCST), the Tower of London task (ToL), and the Reading-the-mind-in-the-eyes-test (RMET). Statistical analysis was performed with the analysis of variance to compare the cognitive performances between BPD patients and controls. A mediation analysis was conducted with the Sobel Test in the BPD patients group. The significance level was *p* ≤ 0.05. (3) Results: significant differences between the two groups were found for several parameters of all the cognitive tests examined: BCST, IGT, ToL, and RMET. Mediation analysis with the Sobel test demonstrated that the percentage of correct answers in the BCST (BCSTc) and the RMET score significantly mediated the relation between the CTQ total score and BPDSI total score. (4) Conclusions: BPD patients showed an impairment of the following executive functions: set shifting, decision making, planning and problem solving, and social cognition abilities, in comparison with controls. Our results suggested that the effect of early trauma on BPD psychopathology was mediated by a deficit in two cognitive domains: cognitive flexibility and social cognition.

## 1. Introduction

Borderline personality disorder (BPD) is a severe mental disorder characterized by a pervasive pattern of instability in affective regulation, interpersonal relationships, self-image, and impulse control which leads to emotional dysregulation, impulsive aggression, repetitive self-injuries, and suicidal tendencies [1,2].

In the last decade, a growing number of investigations and systematic reviews have been focused on potential risk factors that contribute to BPD development. Environmental, temperamental, psychopathological, and neurobiological factors associated with BPD onset were identified [3,4,5,6,7,8]. Current developmental models of BPD indicate that the effects of predisposing factors express through risk processes that unfold across adolescence and implicate interactions between a genetic vulnerability and a harsh, invalidating family environment [9,10].

So, early traumatic experiences in terms of precocious emotional, sexual, and/or physical abuse and neglect, bully victimization, abnormalities in familial behaviors and parent–child relationships, and severe maternal psychopathology have a remarkable role in inducing the onset of borderline psychopathology [7,11,12,13,14,15,16,17].

Although the effects of early traumas on neurocognitive functions are still debated and have yet to be defined, some authors suggested that patients with a higher degree of physical and psychic traumatic experiences in childhood and adolescence show more severe neurocognitive deficits [18]. A hypothesis could be that traumas may influence cognitive capacity as a result of experience-mediated damage in brain structure and function [19].

Studies that have investigated neurocognitive alterations in BDP patients identified patterns of deficits that may be considered rather specific for this disorder and suggested that the severity of neuropsychological abnormalities is associated with a negative clinical outcome [20,21,22], poor treatment adherence [23,24], a higher use of psychiatric services, and higher rates of hospitalization [18]. Among cognitive deficits, the impairment of executive functions and social cognition is considered a prominent feature of BPD patients [9,20,25]. Executive functions include cognitive activities such as making decisions, planning actions, and establishing objectives and motor outputs adapted to external demands [26,27,28]. In BPD patients, significant deficits were found in working memory processes, activities of planning and problem solving, set shifting, and decision making [21,24,29,30,31]. Patients with BPD showed a tendency to make risky choices and are unable to improve their performance and learn from negative feedback in comparison with healthy controls [32].

Another cognitive domain that requires assessment in these patients is social cognition. Social cognition is the set of skills that enables the construction of mental representations of existing relationships between the self and others and the use of these representations to carry out purposeful and context-adapted behaviors. Decoding the mental states of other individuals is related to the ability to decipher signals—for example, facial expressions—that express thoughts and affective states [33,34,35]. Social cognition is one of the impaired cognitive domains in BPD. It is related to the mentalization deficit characteristic of these patients and is clinically expressed with a severe instability of interpersonal relationships.

The way in which traumatic events and neurocognitive deficits contribute to BPD psychopathology and their mutual interactions are still understudied. This study has a twofold objective: (1) to investigate the differences in neurocognitive performances between a group of BPD patients and a group of healthy controls; (2) to verify in a sample of BPD patients whether neurocognitive deficits have the role of mediating the effect of traumas on BPD psychopathology.

## 2. Materials and Methods

### 2.1. Participants

The present study included 69 subjects: 38 outpatients with a diagnosis of BPD according to the criteria of the DSM-5 [1] and 31 healthy controls. All participants were aged between 18 and 60 years. Both groups included males and females. The patients were enrolled from outpatients attending the Center for Personality Disorders of the Department of Neuroscience, University of Turin, Italy. Healthy subjects were recruited among the general population and were matched for gender and age. The diagnosis of BPD was made by an expert clinician (P.B.). All subjects were tested with the Structured Clinical Interview for DSM-5 Clinical Version (SCID-5-CV) and Personality Disorders Version (SCID-5-PD) [36,37] to confirm the diagnosis and exclude other psychiatric disorders. 

The study was carried out in accordance with the Declaration of Helsinki of 1995 (as revised in Edinburgh in 2000) and approved by the Local Ethical Committee (Approval ID 0094867-b). The patients and controls tested in this study were recruited from the sample of a previous trial registered in The Australian and New Zealand Clinical Trial Registry (ACTRN12619000078156). For all patients, written informed consent was obtained prior to their participation. The study followed the rules on the handling of biomedical data (Council of the EU: Data Protection, 2015).

The exclusion criteria were: (1) a lifetime diagnosis of delirium, dementia, amnestic disorder, or other cognitive disorders; schizophrenia or other psychotic disorders; bipolar disorder; ADHD; post-traumatic stress disorder; other personality disorders; (2) a concomitant diagnosis of a major depressive episode; and (3) the occurrence of substance use disorder in the twelve months before evaluation. The patients included in the study received treatment as usual (TAU) in accordance with the guidelines for the treatment of BPD [38,39,40,41,41,42,43,44]. The TAU included mood stabilizers (valproic acid, lamotrigine, and topiramate) and second/third-generation antipsychotics (olanzapine, aripiprazole, and quetiapine).

### 2.2. Assessment

Sociodemographic and clinical variables were registered with a semi-structured interview. Anamnestic reports were confirmed, when possible, by family members or caregivers. Data were entered in a password-protected database.

BPD patients were tested with the Borderline Personality Disorder Severity Index (BPDSI) [45] and the Childhood Trauma Questionnaire–Short Form (CTQ-SF) [46]. All subjects were evaluated with a neurocognitive battery including the Iowa Gambling task (IGT) [47], the Berg card sorting test (BCST) [48], and the Tower of London task (ToL) [49]. In order to assess social cognition, we used the Reading-the-mind-in-the-eyes test (RMET) [50,51].

The BPDSI-IV is a semi-structured interview based on DSM-IV BPD criteria and yields a quantitative index of the current severity and frequency of specific BPD manifestations. The interview consists of 70 items, arranged in nine subscales representing the nine DSM-IV BPD-criteria. For each item, the frequency of the last three months is rated on an 11-point scale, running from 0 (never) to 10 (daily). Identity disturbance items are an exception, since they concern a stable sense of self over a time period rather than a quantifiable symptom. Therefore, identity disturbance items are rated on a scale from 0 (absent) to 4 (dominant, clear, and well defined not knowing who he/she is); the mean score is then multiplied by 2.5. The total score is the sum of the nine averaged criteria scores (range 0–90). The index, but also the separate criteria, possess adequate reliability as well as discriminant, concurrent, and construct validity both in the original version [45] and in the Italian translation [52].

In order to evaluate the presence and severity of childhood trauma, the Childhood Trauma Questionnaire–Short Form (CTQ-SF) was administered. The Childhood Trauma Questionnaire-Short Form (CTQ–SF) is the most widely used retrospective measure for the assessment of early traumatic experiences. It is an easier and more rapid questionnaire developed from the original 70-item Childhood Trauma Questionnaire (CTQ) [53]. It is made of 28 items. Twenty-five of them were retained from the original CTQ and measured experiences of five different types of childhood traumas: emotional abuse, physical abuse, sexual abuse, emotional neglect, and physical neglect. Three additional items provide information on patients’ tendencies toward minimization and negation [54]. For each item, the participant assigns a frequency from never true (1) to very often true (5). Then, the expressed frequencies are converted by the clinician into numerical values of 1 to 5 (or 5 to 1 for inverse-R scoring items). These scores are summed for each of the five clinical scales. The total scores for each scale range from 5 to 25 and provide a quantitative index of trauma severity. The minimization/neglect scale is an exception because it consists of three items (items 10, 16, and 22), and one point is awarded for each item that has been valued 5 (most often true). The total score on the minimization/negation scale is in the range of 0–3. CTQ total scores have also been calculated, since they have been used in previous studies [55,56,57,58]. The sum of subscale scores results in a total score ranging from 25 to 128.

Neurocognitive tests were derived from the PEBL test battery, a freely downloadable and modifiable software [59].

1. The Iowa Gambling task (IGT) evaluates hot cognitive functions [60], particularly decision making [61,62]. The Iowa Gambling task (IGT) is a psychological task thought to simulate real-life decision making. Four virtual decks of cards are presented on a computer screen. Participants are instructed that the cards from each deck will either reward or penalize them. The goal of the game is to win as much money as possible. The decks differ from each other in terms of the balance of reward versus penalty cards. Thus, some decks are more risky (decks A and B), while other decks are less risky (decks C and D), as some decks will tend to cause losses more often than others. The relative sums of the disadvantageous and advantageous decks are subtracted from each other to define the magnitude of deck preference in terms of gain: (deck C + deck D) − (deck A + deck B). This index corresponds to IGT-net. Higher values signify the better performance on the task [63].

In addition, the different choice of decks is evaluated according to the frequency of punishments: decks B and D receive punishments less frequently, while A and C receive them more assiduously. This evaluation is performed by means of the following calculation: (deck B + deck D) − (deck A + deck C). Higher values indicate a propensity towards less frequent losses [63].

2. The Berg card sorting test (BCST): The PEBL version of the Wisconsin card sorting test is used to evaluate cognitive flexibility and set-shifting ability [61]. It is a measure of cool executive functions. The outcomes considered for the neurocognitive assessment of patients and controls are: correct answers (expressed in %); incorrect answers (expressed in %); perseverative errors (expressed in %); non-perseverative errors or set loss (expressed in %); a failure to maintain the set (loss of the correct rule of order during the execution) [64]. It should be specified that, within non-perseverative errors, a subdivision should be made: effective errors should be distinguished from casual errors. Effective errors are non-perseverative and unavoidable errors that are needed to acquire an efficient use of information in order to perform a correct set shifting; in the case of healthy subjects, they occur immediately after the rule change.

3. The Tower of London task (ToL) is used to evaluate any deficit in terms of planning (the organization of a sequence of actions oriented toward a goal), as well as to offer a measure of the ability to perform correct problem solving (acquisition of heuristic strategies to build as many towers in the shortest time possible) [61,65]. The outcomes considered in the Tower of London test are the average number of moves it takes the test subject to solve the problem and the time, expressed in ms, needed to solve it [21,22].

4. The Reading-the-mind-in-the-eyes test (RMET) has been widely used to assess the theory of mind or the ability to recognize the thoughts and feelings of others. This test includes 36 photographs of male and female eyes depicting emotional states. For each photograph, participants are asked to choose the emotional state that best describes the eye expression, choosing between one of four possible emotions. The sum is given by the number of correct answers (maximum 36).

### 2.3. Statistical Analysis

Statistical analyses were performed with the Statistical Package for the Social Sciences, SPSS, version 28 for Windows (SPSS, Chicago, IL, USA).

We performed a *t*-test and chi-square test for demographical variables to exclude significant differences between BPD patients and healthy controls. One-way analysis of variance (ANOVA) was calculated in order to compare the cognitive performances achieved in the four tests of this study (IGT, BCST, ToL, and RMET).

In the group of BPD patients, the Sobel Test (Z) was calculated to investigate the effect of mediation of cognitive deficits between early traumatic experience (CTQ-SF score) and BPD symptoms (BPDSI total score). Mediation occurs when the following conditions are met: (1) the independent variable (IV) significantly predicts the dependent variable (DV); (2) the IV significantly predicts the potential mediator (M); (3) the M predicts the DV; (4) the effect of IV on DV is reduced when the M is included in the model. To determine whether this attenuation was significant and to determine whether M fully or partially mediated the relationship between the IV and the DV, the Sobel test for indirect effects was employed. In this study, numerical values are presented as the mean ± standard deviation (SD) unless otherwise specified. The significance level was *p* ≤ 0.05.

## 3. Results

Sixty-nine subjects were included in the study: 38 outpatients with a diagnosis of BPD (26 women and 12 men) and 31 healthy controls (21 women and 10 men).

The mean age was 34.40 ± 13.54 in the BPD patients and 33.45 ± 11.69 in the controls. The mean age of education was 13.37 ± 2.33 years in the patients group, while it was 15.39 ± 2.04 years in the controls group.

A comparison of sociodemographic data between the two groups was performed with a t test for continuous variables and a Chi-square test for categorical variables. No significant differences were found. The results are displayed in Table 1.

To compare the cognitive performances of patients with BPD and healthy controls, we used one-way ANOVA. A significant difference between the two groups was found for BCSTc (*p* < 0.001; F = 41.575), BCSTe (*p* < 0.001; F = 41.387), BCSTep (*p* = 0.035; F = 4.642), CD-AB (*p* < 0.001; F = 30.194), IGTnet (*p* < 0.001; F = 17.894), ToL Steps (*p* = 0.003; F = 9.318), ToL Time (*p* < 0.001; F = 12.898), and RMET (*p* < 0.001; F = 68.398). We reported the effect size of the ANOVA analysis (η^2^). The results are reported in Table 2.

**Figure 1:**
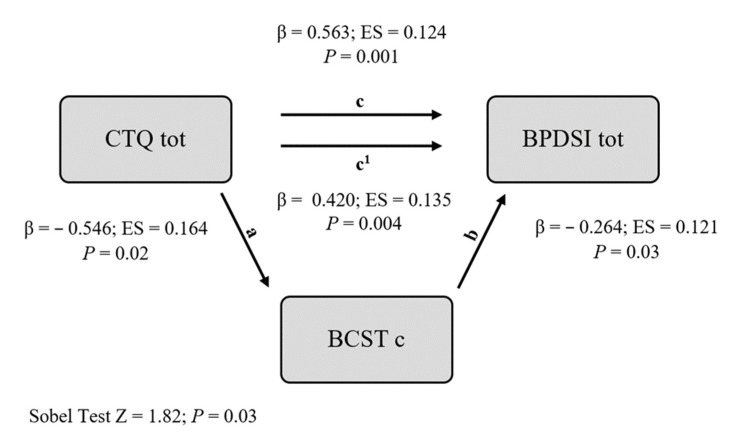
Mediational model (M = BCSTc).

**Figure 2:**
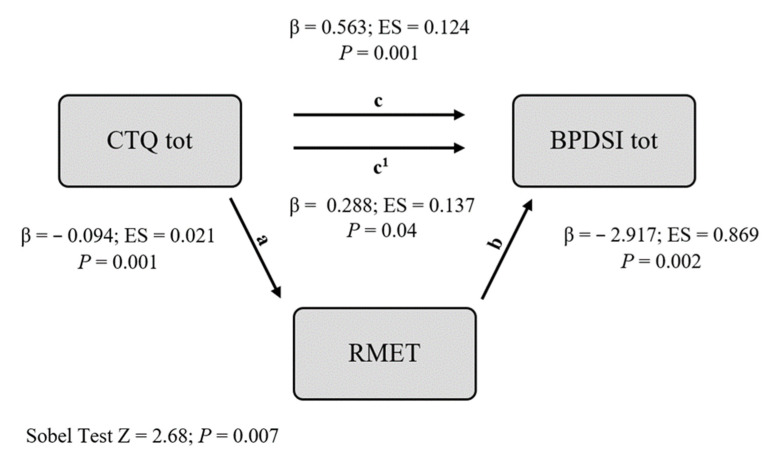
Mediational model (M = RMET).

For each assessment instrument of cognitive performance, we made a mediational hypothesis testing as a mediator between early traumas and BPD symptoms, the parameter that presented the highest mean difference between groups in the one-way ANOVA. We report the results of the two mediational hypotheses for which the Sobel test indicated a significant effect of mediation (BCST and RMET). The Sobel test for IGT and ToL did not reach statistical significance. In our analysis, the IV was the CTQ tot score, the hypothetical M was a measure of cognitive deficit, and the DV was the severity of BPD symptoms. The results are presented in Table 3 and Table 4 and in Figure 1 and Figure 2.

In the first analysis, we tested the effect of the CTQ total score on the BPDSI total score, with BCSTc as a potential mediator (Table 3, Figure 1). The direct effect (c) of the CTQ total score on the BPDSI total score was significant, showing that more severe early traumatic experiences predicted a higher severity of psychopathology (β = 0.563, SE = 0.124, *p* = 0.001). Moreover, the CTQ total score significantly predicted BCSTc (a), with more severe traumas predicting a lower percentage of correct answers in the test of cognitive flexibility (β = −0.546, SE = 0.164, *p* = 0.02). In addition, BCSTc predicted the BPDSI total score (b), with a lower number of correct answers associated with a higher severity of symptoms (β = −0.264, SE = 0.121, *p* = 0.03).

The direct effect of the CTQ total score remained significant after the inclusion of BCSTc as a mediating variable, while it decreased somewhat in magnitude (β from 0.563 to 0.420). 

The Sobel tests for mediation demonstrated that BCSTc significantly mediated the relation between the CTQ total score and BPDSI total score (z = 1.82, *p* = 0.03).

In the second mediation analysis, we tested RMET as a potential mediator of the effect of the CTQ total score on the BPDSI total score (Table 4, Figure 2). The CTQ total score significantly predicted RMET (a), with more severe early traumas associated with a lower ability to recognize the thoughts and feelings of others (β = −0.094, SE = 0.021, *p* = 0.001). Furthermore, the RMET score significantly predicted the BPDSI total score (b), with a lower performance in social cognition associated with more severe BPD symptoms (β = −2.917, SE = 0.869, *p* = 0.002).

Also in this mediation analysis, the direct effect of the CTQ total score on the BPDSI total score remained significant after the inclusion of RMET as a mediating variable, while it decreased somewhat in magnitude (β from 0.563 to 0.288).

The Sobel tests for mediation demonstrated that RMET significantly mediated the relation between the CTQ total score and BPDSI total score (z = 2.68, *p* = 0.007).

## 4. Discussion

The present study aimed to assess the differences in the functioning of specific cognitive domains between a group of BPD patients and a group of healthy controls. Moreover, in the patients group, we set out to evaluate, by a mediation analysis, whether the effect of early traumatic experiences on the psychopathology of the disorder might be partly mediated by deficits in cognitive functions.

As for the first point, our results showed a significant difference in all cognitive domains between the patients and controls. In particular, BPD patients had an impairment of the following executive functions: set shifting (BCST), decision making (IGT), and planning and problem solving (ToL). Although the interest in cognitive deficits in borderline pathology is fairly recent and available studies are still limited, these findings are in line with those of previous investigations [22,24,32,65,66,67,68,69,70,71]. Another interesting result of this study, in accordance with previous investigations, was that the patients group presented significantly impaired social cognition abilities (RMET) in comparison with the controls. In recent years, clinical research paid increasing attention to the social cognitive dysfunctions of patients with BPD, and a growing number of studies have indicated that these patients show significant deficits in the domain of social cognition [72,73,74].

Regarding the second objective of the present study, namely, the hypothesis that certain cognitive deficits act as mediators of the effect of early trauma on borderline psychopathology, it is not possible to compare our findings with the data of preceding studies. To the best of our knowledge, this is the first study to evaluate, by mediation analysis, the complex interactions between early trauma, cognitive impairments, and BPD symptoms. Fairly consistent data are available in the literature on the relationship between early traumatic experiences and the severity of BPD psychopathology [7,8,18,75,76], while less studied and more controversial are the relationships between trauma and neurocognitive deficits [18,77] and between the latter and BPD symptoms [2,18].

Our results suggested that the effect of early trauma on BPD psychopathology was mediated by deficits in two parameters of cognitive domains: the cognitive flexibility or set shifting (measured with the BCST percentage of correct answers - BCSTc) and the social cognition (measured with the RMET score).

These results were reported for the first time and need to be replicated, but in our opinion, they deserve to be carefully considered. In the first case, it can be hypothesized that the multiple traumatic events that occurred at an early age affected cognitive development by making the subject less flexible and thus less capable of adapting to the environmental context and of choosing appropriate strategies in response to different challenges. Deficits in cognitive flexibility and set shifting could be partly responsible for characteristic symptoms of DBP such as the difficulty in controlling anger and impulsivity and the failure to maintain stable relationships across changing situations.

The second result obtained in the mediation analysis is also of considerable interest, as the impairment of social cognition is a key factor in patients with a diagnosis of BPD [2,78,79]. In fact, theories of the development of BPD point out that traumatic events in childhood and adolescence can interfere with the normal development of social cognition and mentalization capacity [79]. A possible interpretation of our finding may be that early and repeated experiences of emotional and physical abuse or neglect cause or provoke a condition of cognitive isolation in which subjects are not able to acknowledge others’ beliefs and affective states. Deficits in empathic abilities and the interpersonal communication of cognitive and affective states can generate or exacerbate BPD symptoms, especially in terms of unstable relationships and uncontrolled reactions without an evaluation of their consequences.

The fact that no significant effects of mediation were found for other cognitive evaluation instruments—in particular, the IGT (to assess the function of decision making) and the ToL (to measure the abilities of planning and problem solving)—is rather difficult to interpret and requires further investigations.

The results of the present study, which underline the role of cognitive domains in BPD pathology, if confirmed, may have useful therapeutic implications. Some authors concluded that it is not enough to obtain symptomatic improvement in order to produce significant effects on overall functioning [80]. Therefore, cognitive deficits should also become a specific target of treatment. For example, cognitive remediation or psychotherapeutic interventions, such as interpersonal psychotherapy or mentalization-based therapy, could produce positive changes in cognitive flexibility, social cognition, and empathy. In addition, preliminary evidence highlighted the opportunity to restore cognitive deficits in BPD patients with noninvasive brain stimulation interventions [81].

Our study suffers from some limitations. The first limit is due to the rather small sample size. A more adequate sample size could be achieved in a multicenter study. A second limitation is related to the predominance of the female gender in the sample. The unequal gender distribution can be a bias, since some authors believe that deficits of cognitive functions are different in males and females. The third limit concerns the mean age of patients, which is rather high considering the age at the onset of personality disorders. It implies that patients are evaluated after a prolonged duration of illness. The fourth limit is the lack of specific mediating analyses, taking in consideration the nine BPDSI subscales rather than the total score. Another limitation is due to the fact that the effects on cognitive functions in the patients group can be partly induced by the treatment received by these subjects, although the medications used in our sample are recent drugs with a relatively low impact on cognition.

## Figures and Tables

**Table 1:** Comparison (with a *t* test and χ^2^ test) of the baseline values of demographic variables between the BPD and healthy control groups ^a^.

Variable	BPD Patients	Healthy Controls	*t*/χ^2^	*p*
Age, y	34.40 ± 13.55	33.45 ± 11.69	0.31	0.76
Men/women, *n*	12/26	10/21	0.01	0.95
Level of education, y	13.37 ± 2.33	15.39 ± 2.04	−3.78	0.33

^a^ Values are the mean ± SD unless otherwise noted. Abbreviations: BPD = borderline personality disorder.

**Table 2:** Comparison (with ANOVA) of the baseline values of measures of cognitive domains between the BPD and healthy control groups ^a^.

Measure	BPD Patients	Healthy Controls	F	*p*	η^2^
RMET	21.58 ± 2.40	25.84 ± 1.73	68.398	<0.001	0.51
BCSTc	57.29 ± 17.10	79.56 ± 9.70	41.575	<0.001	0.38
BCSTe	43.01 ± 17.45	20.44 ± 9.70	41.387	<0.001	0.38
BCSTep	17.65 ± 10.93	12.52 ± 8.30	4.642	0.035	0.07
CD-AB	−9.55 ± 23.27	23.48 ± 26.65	30.194	<0.001	0.31
IGTnet	−680.66 ± 729.12	101.61 ± 805.16	17.894	<0.001	0.21
ToLSteps	7.33 ± 1.13	6.60 ± 0.76	9.318	0.003	0.12
ToLTime (ms)	31,615.06 ± 15,405.61	29,423.11 ± 8807.56	12.898	<0.001	0.16

^a^ Values are the mean ± SD unless otherwise noted. Abbreviations: BCSTc = Berg card sorting test correct answers; BCSTe = Berg card sorting test incorrect answers; BCSTep = Berg card sorting test perseverative errors; IGTnet = Iowa Gambling task net scores; RMET = Reading-the-mind-in-the-eyes test; ToLSTEPS = Tower of London Steps; ToLTIME = Tower of London Time; ms = milliseconds.

**Table 3:** Mediational model (M = BCSTc).

	β	SE	*p*
REGRESSION (a)			
CTQ tot → BCSTc	−0.546	0.164	0.02
REGRESSION (b)			
BCSTc → BPDSI tot	−0.264	0.121	0.03
REGRESSION (c)			
CTQ tot → BPDSI tot	0.563	0.124	<0.001
REGRESSION (c1)			
CTQ tot → BPDSI tot	0.420	0.135	0.004
Sobel Test			
		Z = 1.82	*p* = 0.03

Abbreviations: M = mediator; BCSTc = Berg card sorting test correct; BPDSI tot = Borderline Personality Disorder Severity Index Total Score; CTQ tot = Childhood Trauma Questionnaire total score.

**Table 4:** Mediational model (M = RMET).

	β	SE	*p*
REGRESSION (a)			
CTQ tot → RMET	0.563	0.124	<0.001
REGRESSION (b)			
RMET → BPDSI tot	−2.917	0.869	0.002
REGRESSION (c)			
CTQ tot → BPDSI tot	0.563	0.124	<0.001
REGRESSION (c1)			
CTQ tot → BPDSI tot	0.288	0.137	0.04
Sobel Test			
		Z = 2.68	*p* = 0.007

Abbreviations: M = mediator; BPDSI tot = Borderline Personality Disorder Severity Index Total Score; CTQ tot = Childhood Trauma Questionnaire total score; RMET = Reading-the-mind-in-the-eyes test.

## Data Availability

Our local ethics committee does not allow us to make our sets of data available.
